# Long-term survival rates of tapered self-tapping bone-level implants after immediate placement: a positional effective rationale

**DOI:** 10.1186/s40902-024-00428-7

**Published:** 2024-05-10

**Authors:** Yoon Thu Aung, Mi Young Eo, Buyanbileg Sodnom-Ish, Myung Joo Kim, Soung Min Kim

**Affiliations:** 1 Department of Oral and Maxillofacial Surgery, School of Dentistry, Dental Research Institute, Seoul National University, 101 Daehak-Ro, Jongno-Gu, 03080 Seoul, Republic of Korea; 2 Department of Prosthodontics, School of Dentistry, Dental Research Institute, Seoul National University, Seoul, Republic of Korea

**Keywords:** Immediate implant, Survival rates, Tapered, Sand-blasted, Acid-etched internal submerged dental implants, Positional rationale, Marginal bone loss (MBL)

## Abstract

**Background:**

Immediate implant placement has gained popularity due to its several advantages. However, immediate placement has its challenges, including concerns about primary stability and bone formation around the implant. The aim of the present study is to evaluate the clinical outcomes of tapered, sand-blasted, and acid-etched internal submerged dental implants in various regions of the jaw bones and to provide a positional rationale for immediate implant placement.

**Methods:**

Between 2009 and 2018, a single surgeon at Seoul National University Dental Hospital in Seoul, Korea, immediately inserted 49 dental implants with tapered bone-level design after extraction, in a total of 34 patients. The clinical outcomes were collected and evaluated, focusing on location of implant placement and marginal bone loss (MBL), with consideration of other parameters such as implant diameter and length.

**Results:**

Of 49 immediately installed Luna® (Shinhung Co., Seoul, Korea) dental implants, 23 were placed in the mandible, and 26 were set in the maxilla. The mean age of patients at the time of installation was 65.91 years, ranging from 40 to 86 years. The average follow-up period was 7.43 years, with a range of 5 to 14 years. After a 5-year retrospective evaluation of tapered, sand-blasted, and acid-etched internal submerged dental implants for immediate implant placement, the cumulative survival rate was 93.88%, with 100% survival rate in the mandible and premolar region of both the maxilla and mandible.

**Conclusions:**

After a 5-year evaluation, tapered, sand-blasted, and acid-etched internal submerged dental implants demonstrated good efficacy for immediate placement in various locations within the dental arches, exhibiting effective clinical performance.

## Background

Immediate implant placement is defined as installation of a dental implant immediately following tooth extraction (Third ITI consensus statement) [1]. Immediate implant placement offers advantages such as reduced treatment time, fewer surgical procedures, optimal utilization of existing bone, and high patient satisfaction. The limitations of immediate implant placement include difficulty in achieving primary stability due to the morphology of the extraction socket and the possibility of insufficient bone formation around the implant. Advancements in implant surface treatment technology, however, enable immediate placement of implants with reliable primary stability and favorable clinical outcomes. Sand-blasted, large-grit, acid-etched (SLA)-surfaced implants feature a rough surface. This micro-roughness enhances osseointegration, reduces healing time, and increases biocompatibility [2]. According to the literature, the superior effectiveness of SLA-surfaced implants has been demonstrated, both in healthy individuals and in patients with systemic diseases, particularly when placed in healed sites [3–5]. A retrospective study by Arlin et al. showed higher survival (97.4% vs 95%) and success rates (96.1% vs 93.4%) and lower marginal bone loss (2.6 mm vs 8.9 mm) of SLA surfaced implants compared to titanium plasma spray (TPS)-surfaced ones [6]. The superior survival rate of SLA-surfaced implants (99.1%) compared to resorbable blasting media-surfaced ones (95.2%) was demonstrated by a retrospective study conducted at New York University by Ahmed et al. in 2014 [7].

The purpose of the study was to evaluate the clinical outcomes of tapered, sand-blasted, and acid-etched internal submerged dental implants (Luna®, Shinhung Co., Seoul, Korea) immediately placed in various locations in the oral cavity and to provide the positional rationale for the technique. In this study, we analyzed the clinical and radiographic data of a cohort of patients who underwent immediate implant placement over a 5-year period.

## Methods

Between 2009 and 2018, 49 dental implants were immediately placed after extraction using tapered, sand-blasted, and acid-etched internal submerged dental implants in 34 patients at the Department of Oral and Maxillofacial Surgery, Seoul National University Dental Hospital, Seoul, Korea, by one surgeon (S.M.K). The study protocol and access to patient records were approved by the Institutional Review Board of Seoul National University (IRB No. S-D20200007), Seoul, Korea. The collected and evaluated data included patient information such as age, sex, presence of systemic diseases, and mean follow-up period, and implant-related data, such as the location of implant installation, implant length, and diameter.

### Inclusion criteria

- Patients rehabilitated with a tapered, sand-blasted, and acid-etched internal submerged dental implants (Luna®, Shinhung Co., Seoul, Korea) in the maxillofacial unit of SNUDH
- Patients who received immediate implant placement at any region of the dental arch
- Patient attending regular follow-up with panoramic radiograph for more than 5 years

### Exclusion criteria

- Patients who failed to attend regular follow-up
- Patients who did not have a panoramic radiogram for marginal bone level (MBL) evaluation

### Surgical procedures

Implant installations were performed under local anesthesia after obtaining informed consent from the patient. Flapless, atraumatic tooth extraction was preferred and applied in most cases, preserving existing hard and soft tissue. For multi-rooted teeth, tooth sectioning was performed to maintain the interradicular septum and buccal plate. The socket was debrided with a surgical curette and a copious amount of saline irrigation. Each extracted socket was examined for the available bone apically and laterally to achieve primary stability. The tapered, sand-blasted, and acid-etched internal submerged dental implants were positioned three-dimensionally and installed according to manufacturer guidelines. For anterior teeth, the implant fixtures were installed slightly palatal to the socket, maintaining a buccal wall thickness of at least 1.5 mm. The fixtures in the posterior teeth were positioned in the middle of the interradicular septum parallel to the central fossa of the adjacent teeth. In addition, the immediate implants were installed deeper than implants placed in healed sites. The length and dimeter were determined based on location of implant placement and available bone height and width. Bone grafting procedures were performed depending on the gap between the fixture and the surrounding bone. Sutures of 4–0 polyglactin 910 Vicryl® (Johnson & Johnson, New Brunswick, NJ, USA) were applied with proper tension to maintain buccal bone convexity in cases of bone grafting or without tension in case of no bone grafting. For a platform-switching bone-level implant, re-entry procedures were performed after 3–6 months of installation. Two to 3 months after re-entry, the prosthesis was delivered.

### Assessment and analysis

Patient data that influenced implant survival and marginal bone loss were collected and analyzed, including age, sex, presence systemic diseases, location of implant installation, and implant length and diameter. The success, survival, and failure of the implant were evaluated using The International Congress of Oral Implantologists Pisa Consensus health scale for dental implants [8].

The success of an implant was determined based on the following criteria:

- No pain or tenderness upon function
- No implant mobility
- Less than 2 mm radiographic bone loss from initial installation
- No history of exudate discharge

Failure of an implant was defined as follows:

- Pain upon function
- Implant mobility
- Radiographic bone loss greater than half of the implant length
- Uncontrolled exudate discharge
- Implant loss

The term “survival” was described as radiographic bone loss between 2 mm and half of the implant body without mobility of the implant.

### Marginal bone loss (MBL) evaluation

MBL was measured on the panoramic radiograms that were taken immediately after the implant fixture installation (T0), 3 months after fixture installation (T1), 3 months, 1 year after functional loading (T2, T3), and 5 years after implant installation (T4). The measurement was performed three times as the distance between the implant platform and the most coronal bone-to-implant contact point on the mesial and distal sides of the installed implants, and the mean value was calculated. All measurements were performed using the PACS calibration system (PiView-Star®, ver. 5.0.1; Infinitt Co., Seoul, Korea), with positive and increased numbers indicating bone loss. The changes in bone level from T0 to follow-up appointments and changes between consecutive appointments were evaluated. Possible radiographic image distortion was compensated for using the following formula: Bone loss = (implant length ÷ radiographic implant length) × radiographic bone loss.

### Statistical analysis

The collected descriptive and quantitative data were analyzed using the IBM SPSS® Statistics software (ver. 26.0; IBM, Armonk, NY, USA). For MBL analysis, mean and standard deviation were calculated. The Shapiro–Wilk test was used to test the normal distribution of the variables. Independent sample *T*-test was performed to identify statistical significance at a *P*-value < 0.05. For survival rate analysis according to location, implant diameter, and length, a one-way ANOVA test was used to determine the statistical significance.

## Results

Of 49 immediately installed implants in 34 patients, 23 were placed in the mandible, and 26 were in the maxilla; 13 implants were immediately installed in the anterior region (9 in maxilla, 4 in mandible), 12 were in the premolar region (8 in maxilla, 4 in mandible), and 24 (48.98%) were installed in the molar region (9 in maxilla, 15 in mandible). The mean age of patients at the time of installation was 65.91 years, ranging from 40 to 86 years. The average follow-up period was 7.43 years, with a range of 5 to 14 years. The most common implants had a diameter of 4 mm or a length of 8.5 mm, accounting for 40.82% and 53.36% of cases, respectively (Table 1).

**Table 1:** Demographic data of patients and implants

		Frequency	Percentage
Patient data	Number	34	100
	Sex (male/female)	13/23	38.24/61.76
	Mean age (range)	65.91 (40–86)	
	Presence of systemic diseases	23	61.76
	Follow-up period (mean ± SD)	7.43 ± 2.72	
Implant data	Number	49	100
	Dental arch		
	Maxilla	26	53.06
	Mandible	23	46.94
	Location		
	Anterior	13	26.53
	Premolar	12	24.49
	Molar	24	48.98

The units for age and follow-up period are years

*SD*, standard deviation

Tapered, sand-blasted, and acid-etched internal submerged dental implants of the Shinhung® Implant System

### Survival rate analysis

After a 5-year retrospective clinical and radiographic evaluation of immediately placed implants, the cumulative survival rate was 93.88%. Implants in anterior, premolar, and molar areas of mandible show 100% survival rate. In the maxilla, implants in the premolar region had 100% survival rate. In contrast, the maxillary anterior region had the lowest survival rate of 85.38%, while the survival rate in the maxillary molar region was 95.83% (Table 2).

**Table 2:** Survival and failure of implants depending on location in the dental arch

	Maxilla (total/fail)	Mandible (total/fail)	Sum (total/fail)	Survival rate
Anterior	9/2	4/0	13/3	85.38
Premolar	8/0	4/0	12/0	100
Molar	9/1	15/0	24/1	95.83
Sum	26/3	23/0	49/3	93.88
Survival rate	88.46	100	93.88	

The descriptive data were analyzed with the IBM SPSS Statistics software (ver. 26.0; IBM, Armonk, NY, USA)

*P*-value < 0.05 was considered statistically significant

Survival rates are not significant among 6 groups of locations (maxillary: anterior, premolar, molar and mandibular: anterior, premolar, molar)

The survival rate of implants with 3.5- and 5-mm dimeter was 100%, while 4-mm and 4.5-mm-diameter implants, installed in 55.2% and 26.5% of respective cases, had survival rates of 96.2% and 84.61% (Table 3). Evaluation of survival rate based on implant length revealed 100% survival in implants with length of 10 mm or 11.5 mm, while one 7-mm and two 8.5-mm implants failed, resulting in survival rates of 87.5% and 93.75%, respectively (Table 4). The survival rates among the 4 respective groups of implant diameter and length are not statistically significant.

**Table 3:** Survival and failure of implants depending on implant diameter

Implant diameter (mm)	Frequency	Percentage	Survival rate
3.5	6	12.2	100
4	27	55.2	96.2 (1 failure)
4.5	13	26.5	84.61 (2 failures)
5	3	6.1	100
Total	49	100	93.88

The descriptive data were analyzed with the IBM SPSS Statistics software (ver. 26.0; IBM, Armonk, NY, USA)

*P*-value < 0.05 was considered statistically significant

Survival rates are not significant among 4 groups of implant diameter

**Table 4:** Survival and failure of implants depending on implant length

Implant length (mm)	Frequency	Percentage	Survival rate
7	8	16.3	87.5 (1 failure)
8.5	32	65.3	93.75 (2 failures)
10	7	14.3	100
11.5	2	4.1	100
Total	49	100	93.88

The descriptive data were analyzed with IBM SPSS Statistics software (ver. 26.0; IBM, Armonk, NY, USA)

*P*-value < 0.05 was considered statistically significant

Survival rates are not significant among 4 groups of implant length

### MBL evaluation

**Fig. 1:**
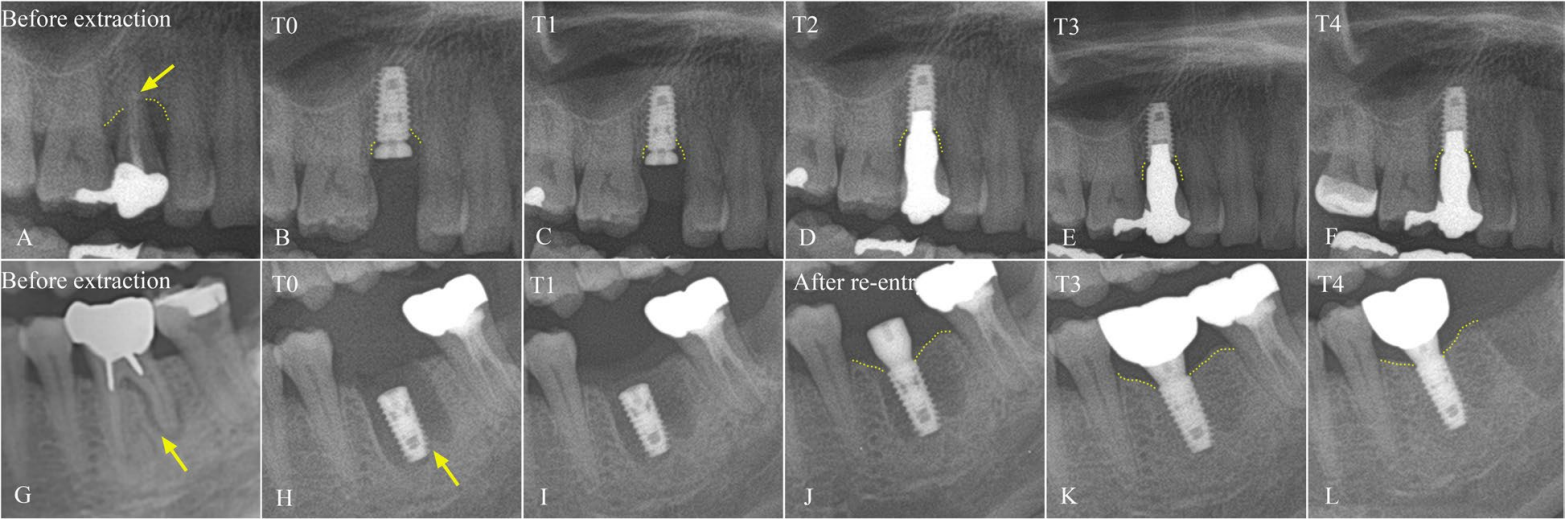
Representative cases of radiographic marginal bone level at maxillary premolar (**A**–**F**) and mandibular molar (**G**–**L**). Panoramic radiograph before extraction of #15 with radiographic bone loss due to chronic periodontitis (**A**). Panoramic radiograph taken immediately after immediate implant installation with 4.5 × 8.5 mm Luna® bone level implant (**B**). Panoramic radiograph at 3 months after implant installation with stable bone level (yellow dots) (**C**). Panoramic radiographs at 3 months and 1 year after prosthesis loading (**D**, **E**) and 5 years after implant installation (**F**) showing stable and insignificantly increased marginal bone level. Panoramic radiograph before extraction of #36 with periodontal ligament space widening due to periodontitis on the distal root (yellow arrow) with sufficient interradicular bone for immediate implant placement (**G**). Panoramic radiograph after immediate installation of 4.5 × 8.5 mm Luna® implant in the interradicular bone (yellow arrow) (**H**). Panoramic radiograph 3 months after implant installation with increased radiographic bone density around implant (**I**). Panoramic radiographs after re-entry procedure (6 months after initial installation) showing increased marginal bone level around implant platform (yellow dots) (**J**) and 1 year after prosthesis loading (**K**) and 5 years after implant installation (**L**) with stable marginal bone level (yellow dots)

Significant marginal bone gain was observed at the mesial surface starting at 3 months after implant installation (− 0.68 ± 1.61 mm), and progressive bone gain on both mesial and distal surfaces was significant at 3 months and 1 year after functional loading until 5 years after installation compared to initial installation (*p* < 0.05) (Table 5). Although the marginal bone level on both mesial and distal sides increased at every appointment compared to the previous measurements, the difference was not statistically significant (*p* > 0.05). Representative cases of marginal bone gain in the panoramic radiographs were shown in Fig. 1.

**Table 5:** Marginal bone level (MBL) evaluation of immediate implants

	MBL compared to initial implant installation T0 (mm)		MBL compared to previous measurements (mm)	
	Mesial	Distal	Mesial	Distal
T1	− 0.68 ± 1.61 (0.04)*	− 0.52 ± 1.10 (0.06)		
T2	− 1.11 ± 1.91 (0.02)*	− 0.89 ± 1.53 (0.02)*	− 0.43 ± 1.41 (0.19)	− 0.36 ± 1.20 (0.40)
T3	− 1.28 ± 1.67 (0.00)*	− 1.01 ± 1.46 (0.00)*	− 0.17 ± 0.88 (0.29)	− 0.12 ± 0.78 (0.23)
T4	− 1.31 ± 1.74 (0.00)*	− 1.05 ± 1.71 (0.00)*	− 0.03 ± 0.87 (0.97)	− 0.04 ± 1.03 (0.98)

*T0*, immediately after implant installation; *T1*, 3 months after installation; *T2*, 3 months after prosthesis loading; *T3*, 1 year after loading; *T4*, 5 years after installation

Negative value indicates bone gain

Number in parentheses is *P*-value

^*^P-value < 0.05 was considered statistically significant

### Evaluation of past medical history

In our study, 61.74% of patients have the underlying metabolic diseases such as hypertension (29.4%), diabetes mellitus (11.76%), cardiovascular diseases (14.7%), and thyroid disease (5.88%), and 8.82% have the bone-related diseases like osteoporosis (5.88%) and multiple myeloma (2.94%) (Table 6).

**Table 6:** Patient’s past medical history

Medical history	Frequency	Percentage
Hypertension	10	29.4
Diabetes mellitus (DM)	4	11.76
Osteoporosis	2	5.88
Cardiovascular diseases (CVD)	5	14.7
HBV, HCV-related hepatitis	3	8.82
Thyroid diseases	2	5.88
Rheumatism	1	2.94
Bullous pemphigus	1	2.94
Gastric cancer	1	2.94
Oral cancer	1	2.94
Multiple myeloma	1	2.94
Renal transplant	1	2.94
HIV	1	2.94
Prostate hypertrophy	1	2.94
Peripheral neuropathy	1	2.94

The percentage was calculated upon 34 patients

### Failure cases evaluation

**Fig. 2:**
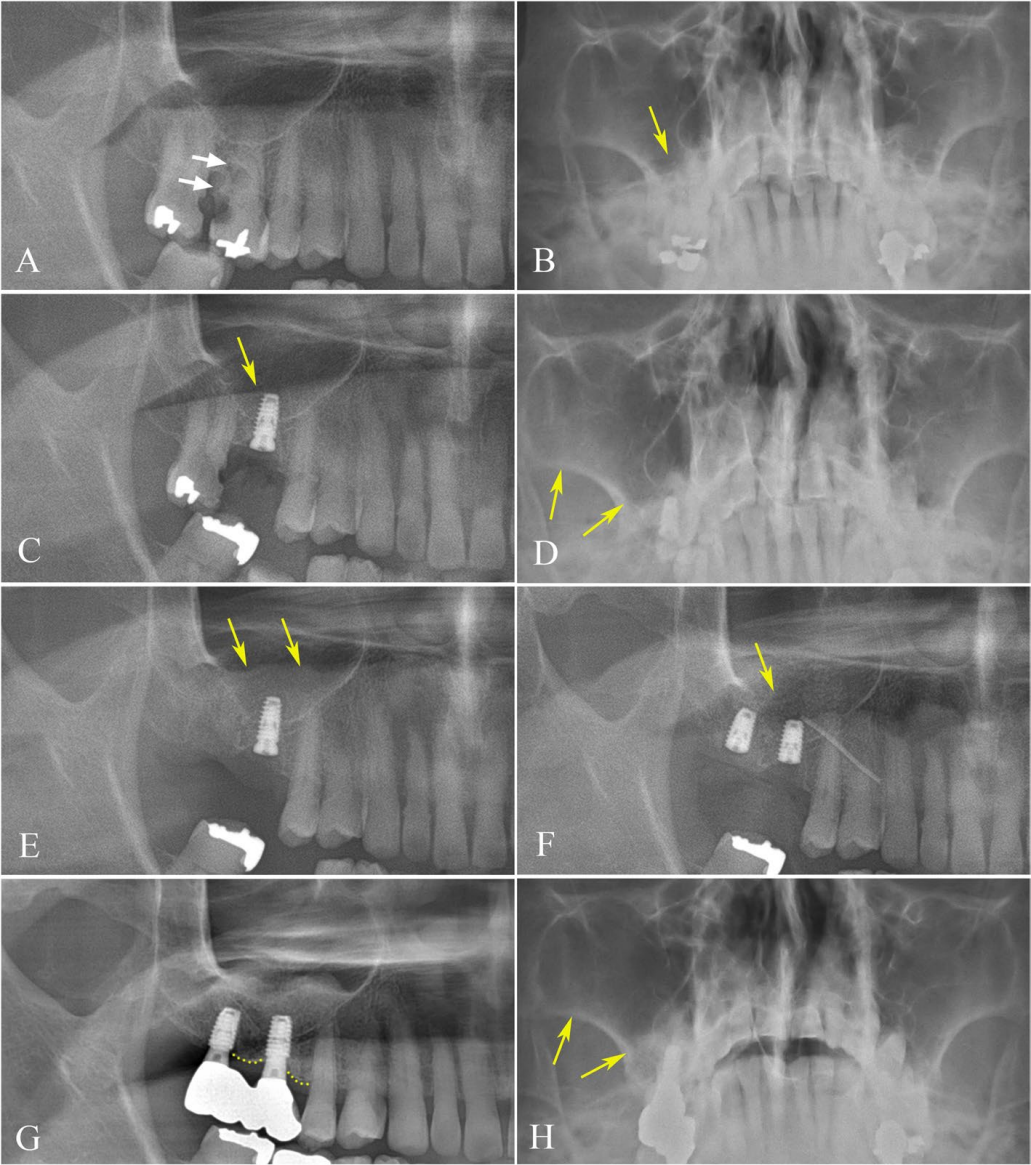
Radiographic views of failure case at #16 (**A**–**H**). Panoramic radiograph before extraction of #16 with distal caries extending to the root (white arrows) (**A**). Pre-operative Waters’ view showing no significant inflammatory changes in the maxillary sinus around #16 (yellow arrow) (**B**). Panoramic radiograph at 1 month after immediate implant placement with no significant changes in sinus mucosa around installed implant (**C**). Waters’ view (**D**) and panoramic view (**E**) at 3 months after implant installation illustrating sinus lining thickening (yellow arrows). Post-operative panoramic radiograph of re-entry procedure at 5 months after initial installation (**F**) showing changed one diameter larger implant (yellow arrow) after removal of inflammatory tissue and sinus lifting with inserted silastic drain. A recent panoramic view (**G**) revealing well-osseointegrated implant, exhibiting no signs of marginal bone loss (yellow dots), and Waters’ view (**H**) illustrating a pristine maxillary sinus (yellow arrows), 6 years after re-installation

Implant failure can be categorized into early and late failure depending on the time of failure. Early failure of an implant is characterized by lack of osseointegration [2, 4]. Among 49 immediately installed implants, 3 implants of 2 patients failed (6.12%). One implant at the #16 area in a patient with history of hypertension and renal transplantation failed (Fig. 2). In this case, the patient complained of symptoms of sinusitis at 3 months after implant installation. Thickening of the mucosa lining was identified on Waters’ view and panoramic view (Fig. 2D, E). Failure of osseointegration was observed with implant mobility at the re-entry procedure performed 5 months after installation. The failed implant was replaced with a 4.5 × 7 mm bone level implant from the same company, inflamed tissue was removed, and sinus lifting was performed (Fig. 2F). The changed implant was in use without problems at the time of final assessment (Fig. 2G, H).

Two implants at the maxillary canine (#13 and #23) of one maxillary edentulous patient who previously used dentures failed and showed clinical signs of peri-implantitis with pus discharge, swelling at the left side, implant mobility, and radiographic bone loss of more than half of the implant length after 9 months and at 1.5 years after initial installation before prosthesis loading, respectively. In this case, there was extensive pneumatization of the maxillary sinuses on both sides, and the patient had a history of maxillary sinusitis and had undergone modified endoscopic sinus surgery on both sides. The failed implant at tooth #13 was replaced with a Straumann BL® 3.3 × 8 mm implant after sinus lifting, and that at tooth #23 was replaced with a Luna® 3.5 × 8.5-mm implant after socket grafting. Both implants were functional with no problem at the time of final assessment.

## Discussion

The current four classification categories based on timing of implant placement after tooth extraction initially were proposed at the third ITI consensus conference and were modified by Chen and Buser in 2018 [1, 9]. Immediate placement of an implant after extraction was classified as type 1 and has several advantages, including reduction of treatment duration and procedures, high patient satisfaction, and optimized use of existing bone. In contrast to healing of the extraction socket with a substantial amount of bone loss, the significant increase in bone level observed 3 months after implant installation on the mesial sides in our study illustrated bone regeneration around immediately installed implants. We also hypothesize that immediate placement of an implant can also decrease alveolar ridge resorption to a variable degree.

Osseointegration is characterized histologically by direct bonding of bone to the implant surface and clinically by fusion of the implant with the surrounding bony structure. Interactions between implant surfaces and bone tissue are crucial for osseointegration [2]. Elias et al. reported that implant shape, size, and surface morphology, as well as surgical technique, all play a significant role [10]. SLA surface is one of the most documented rough surfaces in implantology, demonstrating both macro and micro surface roughness. Abrahamsson et al. [11] in 2004 conducted an in vivo experiment in canine and proved the effectiveness of SLA-surfaced implants with higher bone to implant contact and bone formation around the implant by contact osteogenesis. In a study by Donos et al. in 2011 [12], genes associated with regeneration, such as Notch-1, exhibited increased expression on the SLA surface compared to the machined group, and the Wnt signaling pathway is believed to play a significant role as a regulator in regenerative responses to various titanium surfaces. Several clinical studies have illustrated the clinical effectiveness of SLA-surfaced implants rehabilitated in healing sites [2, 4, 13].

As for the implant size, Heimes et al. concluded on the review in 2023 that the larger diameter increases the contact area and provides better primary stability while the linear relationship between implant length and primary stability ends at 12 mm [14]. We recommended choosing the implant length and diameter according to the position of implant placement, anatomical considerations of the area, space and bone availability, and load distribution. From aspect of shape of implant, tapered implant has the advantages of increased primary stability by exerting lateral compressive force on the cortical bone and better load distribution and osseointegration than straight design, which stabilize implant by transmitting static frictional force along implant axis [14]. In addition, the tapered implant can reduce surgical trauma during implant installation and subsequently increase bone-to-implant contact [15].

The implant used in our study possesses the internal connection with hexagon shape with morse taper, which seems to be more efficient concerning biological aspects, allowing lower bacterial leakage with the best sealing ability and present better result in crestal bone loss than external connections. In addition, the internal connection type shows the advantage of high mechanical stability. According to these authors, the internal morse taper type of connection showed higher reliability in the esthetic region [16].

The study conducted by Sodnom-Ish et al. on the survival rate of 105 tissue-level tapered SLA surface implants, which were placed with delayed implantation in 61 patients, demonstrated a survival rate of 98.1% [4]. The higher survival rate observed for tissue-level implants compared to bone-level implants in this study may be attributed to the uneven distribution of samples and timing of implant placement. However, it is important to note that the survival rate of tissue-level implants with immediate placement remains limited in the current literature.

In MBL analysis of our installation of tapered, sand-blasted, and acid-etched internal submerged implants from Shinhung Implant System, the significant regeneration of bone around the implant at 3 months after implant installation suggests the immediate efficacy of SLA surface on bone formation. Even though one of our previous studies of immediate implantation in the posterior maxilla showed fluctuation in bone regeneration and bone loss [17], a consecutive increase in bone level until 5 years after installation was observed in the present study indicating the medium-term effectiveness of SLA-surfaced implants. Several factors have been identified associated with survival and MBL in a 5-year retrospective study. According to Song et al. [18], MBL during 5 years of implant installation is significantly influenced by factors such as smoking status, type of abutment connection, and implant surface. Marginal bone gain in our study can also be influenced by other factors like bone grafting during implant installation. However, most of the cases (91.8%) have not performed additional bone grafting at the time of implant placement. Rather than additional bone graft, positioning the implant fixture below the adjacent crestal bone level in case of premolar and anterior region. In the molar region, we recommended to follow the bone grafting guideline prescribed by Mustakim et al. [17] depending on the presence of interradicular septum and ABH.

In the assessment of MBL around implant, although cone-beam computed topography (CBCT) has the advantage of 3-dimensional assessment, the application of CBCT for post-operative implant assessment is not generally recommended due to titanium artifact and higher radiation dose compared to 2-dimentional radiograph. On the other hand, based on a study of prediction of dental implant failure from deep learning of periapical and panoramic radiograph [19], the diagnostic accuracy and precision of periapical radiograph was 78.6% and 0.84, while that of panoramic radiograph was 78.7% and 0.87, respectively. Moreover, panoramic radiograph is valuable for patients with multiple implants along the jaws with different timepoints.

Success of an immediate implant depends on the primary stability acquired from apical and lateral bone [17]. The clinical result is highly predictable when the ideal conditions for immediate placement are met. The general considerations for successful immediate implant placement are an extraction socket free of acute infection, atraumatic extraction with a flapless procedure, intact facial bone with sufficient thickness (1.5 mm), availability of > 5 mm basal or palatal bone to allow primary stability, three-dimensional implant positioning in relation to existing socket morphology, a sufficient gap between implant and facial wall (> 2 mm), and choice of implant according to location and availability of bone.

In addition to general considerations, the positional rationale concerning the location of implant placement also needs to be addressed. For immediate implant placement in the maxillary anterior region, factors such as a single tooth socket with thin buccal bone, high esthetic demand, and the relationship with anatomical structures (nasal floor, nasopalatine canal, hyper-pneumatized maxillary sinus) are major concerns. The adequacy of facial bone thickness (≥ 1 mm) and apical bone (≥ 5 mm) should be assessed before deciding on immediate placement. Subsequently, palatal positioning of the implant in relation to the extracted socket, with an at least 2 mm gap between the implant and facial bone with bone graft during flapless extraction enhances esthetic outcomes [20]. A single- or double-rooted maxillary premolar area, particularly when dealing with relatively low bone quality, can pose challenges, especially in cases of maxillary sinus hyperpneumatization. In our present study, the premolar region exhibited significantly high survival rates (100%), emphasizing the importance of adhering to general considerations for implant placement and appropriate choices regarding implant diameter and length.

Immediate placement of implants in the maxillary molar region requires special considerations owing to its multi-rooted nature, low bone density, and proximity to the maxillary sinus. Essential factors to be considered include the presence of an adequate interradicular septum, the remaining alveolar bone height (ABH), and the absence of maxillary sinus infection. It is advisable to perform tooth sectioning during extraction to prevent injury to the interradicular septum. Mustakim et al. [17] have proposed guidelines for immediate implant placement in the maxillary molar region, considering adequate interradicular bone and ABH. The study classified sockets into types I, II, and III based on interradicular bone and types A, B, and C based on ABH. Immediate placement can be performed in type I sockets with adequate interradicular bone, eliminating the need for additional bone grafting. For type II sockets, immediate implant placement with bone grafting is recommended, while use of wide-diameter implants is suggested for a type III interradicular septum with inadequate lateral support. ABH determines the need for sinus lifting, where type A (ABH > 8 mm) allows immediate implant placement without sinus lifting, type B ABH indicates need for sinus lifting or consideration of short dental implant, and type C (ABH < 6 mm) can be suitable for implant installation after sinus lifting.

When contemplating immediate implant placement in the narrow ridge of the anterior mandible, a narrow-diameter implant can be chosen to maintain adequate facial bone width. Consideration of flap design is essential to prevent injury to the mental foramen during implant installation in the premolar region. If a vertical incision is necessary for visualization, it is recommended to place the incision between the mandibular canine and the first premolar. For immediate implant placement in mandibular molars, use of short dental implants in a deeper restoration-guided implant position is recommended to avoid injury to the inferior alveolar nerve. Tooth sectioning, as in maxillary molars, is also advisable for mandibular molars to maintain the interradicular septum for apical primary stability. Our study shows that immediate implant placement has a high survival rate in all regions of the mandible when adhering to the presented considerations.

All three failure cases in our study were associated with maxillary sinus pathology. Our previous study on structural analysis of sinusitis-related implant failure demonstrated metal contaminants on the surfaces of the failed implants. Additionally, inflammatory infiltrates and high osteoclastic activity were observed in the surrounding bone graft materials [21]. The possible etiologies of implant failure in the present study could include pre-existing sinusitis and Schneiderian membrane perforation, insufficient residual alveolar bone height, implant surface contamination, graft material-related reactions, and uneven distribution of occlusal force.

The previous study of implant success and survival rate in 1019 Luna® implants in medically compromised patients revealed the comparable survival rate (97.0%) with those of healthy patients [3]. The review literature by Sbricoli et al. concludes for no association between diabetes, cardiovascular diseases, hypertension, or osteoporosis and the risk of peri-implantitis [22]. This agrees with the population-based study in 6384 patients during 31-year follow-up periods [23]. Among the three failure cases, the patient with one implant failure at #16 has hypertension and received renal transplantation without taking immunosuppressant medication. However, another immediate implant at mandibular molar region of the same patient was functioning without bone loss until the time of assessment.

The limitations of the present study include the retrospective nature and the fact that all surgeries were carried out by a single surgeon. Moreover, future study with the extended follow-up duration, larger sample size and comparative study design with delayed implant placement outcomes could yield valuable insights.

## Conclusions

Within the limited context of the study, the survival rate based on location suggests that immediate implant placement can be reliably conducted in the mandible where bone quality is good, especially in the premolar area (and even in the maxilla), according to our positional rationale. Proper case selection, meticulous surgical planning, and procedures choices are paramount in immediate implant placement, especially in the maxillary anterior and molar areas where failures are more common. Delayed placement with sinus lifting and bone grafting is recommended for the maxillary molar area if there is an association with maxillary sinus pathology. After a 5-year evaluation, tapered, sand-blasted, and acid-etched internal submerged dental implants demonstrated good efficacy for immediate placement in various locations of the dental arches, exhibiting effective clinical performance and continuous bone gain. Through acknowledging the correlation between implant positioning in the maxilla and mandible and their respective survival rates and efficacy, clinicians can exercise prudence in navigating technical and surgical intricacies when placing implant in regions prone to heightened failure rates. Conversely, they can confidently proceed with implant placement in areas boasting elevated rates of implant survival, guided by informed positional deliberations.

## Data Availability

This study was conducted as a retrospective research method, and raw data can be provided upon the request of the journal.

## Abbreviations

MBL: Marginal bone loss
SLA: Sand-blasted, large-grit, acid-etched
ABH: Alveolar bone height
CBCT: Cone-beam computed topography
